# Lamellipodin Is Important for Cell-to-Cell Spread and Actin-Based Motility in Listeria monocytogenes

**DOI:** 10.1128/IAI.00193-15

**Published:** 2015-08-12

**Authors:** Jiahui Wang, Jane E. King, Marie Goldrick, Martin Lowe, Frank B. Gertler, Ian S. Roberts

**Affiliations:** a Faculty of Life Sciences, The University of Manchester, Manchester, United Kingdom; b Department of Biology and Center for Cancer Research, MIT, Cambridge, Massachusetts, USA

## Abstract

Listeria monocytogenes is a foodborne pathogen capable of invading a broad range of cell types and replicating within the host cell cytoplasm. This paper describes the colocalization of host cell lamellipodin (Lpd) with intracellular L. monocytogenes detectable 6 h postinfection of epithelial cells. The association was mediated via interactions between both the peckstrin homology (PH) domain in Lpd and phosphatidylinositol (3,4)-bisphosphate [PI(3,4)P_2_] on the bacterial surface and by interactions between the C-terminal EVH1 (Ena/VASP [vasodilator-stimulated phosphoprotein] homology domain 1) binding domains of Lpd and the host VASP (vasodilator-stimulated phosphoprotein) recruited to the bacterial cell surface by the listerial ActA protein. Depletion of Lpd by short interfering RNA (siRNA) resulted in reduced plaque size and number, indicating a role for Lpd in cell-to-cell spread. In contrast, overexpression of Lpd resulted in an increase in the number of L. monocytogenes-containing protrusions (listeriopods). Manipulation of the levels of Lpd within the cell also affected the intracellular velocity of L. monocytogenes, with a reduction in Lpd corresponding to an increase in intracellular velocity. These data, together with the observation that Lpd accumulated at the interface between the bacteria and the developing actin tail at the initiation of actin-based movement, indicate a possible role for Lpd in the actin-based movement and the cell-to-cell spread of L. monocytogenes.

## INTRODUCTION

Listeria monocytogenes is a Gram-positive, facultative, foodborne intracellular pathogenic bacterium responsible for causing meningoencephalitis, septicemia, gastroenteritis, and abortion in humans, with a high mortality rate (1, 2). Through its intracellular life cycle, L. monocytogenes is able to induce its own uptake into both phagocytic cells (3) and nonphagocytic cells (4–6). Following uptake, it escapes from phagosomes to multiply within the mammalian cell cytosol and exploit host actin polymerization to form a “tail-like” structure, which provides the force to move around within the cytosol and spread to adjacent cells (reviewed in reference 7). The recruitment and polymerization of actin require the L. monocytogenes transmembrane protein ActA (8), which is also required by the bacterium to escape autophagy (9) and in its intestinal colonization and carriage (10).

ActA functions by mimicking the activity of the eukaryotic WASP (Wiskott-Aldrich syndrome protein) family of actin nucleating factors (reviewed in references 11 and 12). ActA contains a VCA (verprolin homology, cofilin homology, and acidic) region at the N terminus, which activates the Arp2/3 complex, critical for actin polymerization (13). In addition to activating Arp2/3, ActA interacts with ATP-G-actin through its actin binding region (14). The central part of ActA contains a poly-proline region with four FPPPP/FPPIP motifs responsible for binding to the EVH1 (Ena/VASP [vasodilator-stimulated phosphoprotein] homology domain 1) domain of VASP to control the geometry of the network formed by the Arp2/3 complex (13, 15). VASP is found at sites of active actin polymerization and is a substrate for cyclic GMP (cGMP)- or cyclic AMP (cAMP)-dependent kinases (16). It can recruit profilin, provide polymerization-competent actin monomers to the N terminus of ActA (13), and interact with F-actin through its C-terminal EVH2 domain, thus providing a linkage of the bacterium to the tail (15). VASP protein is important for facilitating rapid and consistent movement of L. monocytogenes (17).

L. monocytogenes spreads from cell to cell through the generation of bacterial protrusions that are engulfed in the adjacent cell followed by escape into the cytosol of the newly infected cell (11). This is the least-well-understood stage of the intracellular life cycle of L. monocytogenes. Initially, the ERM (ezrin/radixin/moesin) family of proteins was shown to be important in the cell-to-cell spread of L. monocytogenes (18). It was hypothesized that ERM proteins may provide rigidity to these protrusions by cross-linking F-actin tails to the host plasma membrane (18). The L. monocytogenes protein InlC has been shown to interact with the host scaffold protein Tuba, perturbing its interactions with N-WASP and thereby reducing cell surface tension and promoting cell-to-cell spread (19). Recently, it has been shown that inhibition of host cell Cdc42 protein by L. monocytogenes is required for efficient protrusion formation (20). However, there are still many unanswered questions regarding the mechanism by which L. monocytogenes spreads from cell to cell.

One possible candidate for playing a role in cell-to-cell spread is Lpd, which is known to play a critical role in cell migration, mediating lamellipodin formation through regulating actin dynamics (21). The regulation of actin dynamics at the leading edge during cell migration involves a number of positive- and negative-feedback loops, and it is the balance between actin filament branching and elongation that appears critical in lamellipodial persistence (reviewed in reference 22). Previously, Lpd was shown to colocalize with vaccinia virus and enteropathogenic Escherichia coli (EPEC) but not Shigella flexneri or L. monocytogenes 4 h postinfection (23). We wanted to determine if Lpd was associated with L. monocytogenes at later time points following infection of HeLa cells and establish more fully what role Lpd might play in the intracellular life cycle of L. monocytogenes.

In this report, we describe the localization of host cell Lpd to intracellular L. monocytogenes 6 h postinfection. The association was mediated via interactions between Lpd and phosphatidylinositol (3,4)-bisphosphate [PI(3,4)P_2_] and between Lpd and VASP recruited to the bacterial cell surface via ActA. The recruitment of Lpd was essential for efficient cell-to-cell spread by L. monocytogenes, indicating for the first time a role for Lpd in this process. Manipulation of the levels of Lpd within the cell affected the formation of L. monocytogenes-containing protrusions (listeriopods) as well as the intracellular speed of L. monocytogenes motility, indicating a role for Lpd both in the cell-to-cell spread and in the actin-based movement of L. monocytogenes within the cell.

## MATERIALS AND METHODS

### Bacterial strains and culture conditions.

L. monocytogenes serotype 1/2a strain EGDe:InlA^m^ engineered for murine oral infection (24) was used as the wild type, and all mutations were generated in this background. The InlA^m^ mutation has no effect on the ability of this strain to infect human cells *in vitro* (25). L. monocytogenes cells were cultured in tryptone soya broth (TSB; Oxoid), which can be supplemented with 5 μg/ml erythromycin, when plasmids were maintained. L. monocytogenes was grown and reached an absorbance at 600 nm (*A*_600_) of 0.6 at 37°C before infection of cells.

### Molecular cloning and generation of L. monocytogenes mutant strains.

Subcloning and PCR were performed using standard methods. Enhanced green fluorescent protein (EGFP) expression constructs were cloned by amplifying the PH (amino acids [aa] 383 to 535), PH-profilin (aa 380 to 869), profilin (aa 535 to 869), EVHB (aa 861 to 1220), EVHBa (aa 868 to 880), EVHBb (aa 868 to 937), EVHBc (aa 938 to 1062), EVHBd (aa 1063 to 1212), and EVHBe (aa 1201 to 1212) fragments of Lpd plus the ATG start codon using pEGFP-C1hLPD (kindly provided by F. B. Gertler, MIT, USA) as the template and inserted in frame into pEGFP-N2 expression vector. Site-directed mutagenesis of the two key amino acids Lys and Arg for PIP binding in the Lpd-PH domain was performed sequentially as described previously (25) using primers PH-SDM-K-F/PH-SDM-K-R and PH-SDM-R-F/PH-SDM-R-R (see Table S1 in the supplemental material). Phe-Ala or Lue-Ala substitution in all six FPPPP/LPPPP motifs of the Lpd-EVH1 binding domain was achieved by direct DNA synthesis (Invitrogen). In-frame deletions of the *actA* and *actA* EVH binding region (aa 264 to 390) were generated through induced homologue recombination. Around 600 bp of the immediate upstream and downstream regions of the genes to be deleted was amplified by PCR using chromosomal DNA as the template and with primers actAKO1-F/actAKO1-R, actAKO2-F/actAKO2-R, actAEVHBKO1-F/actAEVHBKO1-R, and actAEVHBKO2-F/actAEVHBKO2-R (see Table S1). The amplified PCR products were digested with appropriate restriction enzymes and simultaneously ligated into temperature-sensitive shuttle plasmid pAUL-A (26). After transformation into L. monocytogenes, induced integration of the plasmid at 42°C, and the second recombination event, only the translational start and stop and fewer than 8 codons of each open reading frame remained. Successful generation of mutants was confirmed by colony PCR and subsequent nucleotide determination. Complementation of each mutant was achieved by expression of the cloned wild-type gene on plasmid pUNK1 as described previously (25).

### Cell culture and infection.

HeLa cells (ATCC CCL-2) were maintained in Dulbecco's modified Eagle medium (DMEM; Sigma) containing 10% fetal bovine serum (FBS; Sigma) and incubated at 37°C in humidified air with 5% CO_2_. Where infection with L. monocytogenes was required, semiconfluent cell monolayers were washed twice with prewarmed phosphate-buffered saline (PBS) before infection with mid-log-phase L. monocytogenes in 1 ml serum-free medium was performed at a multiplicity of infection (MOI) of 10:1 for 2 h. After 3 washes, cells were incubated in DMEM–10 μg/ml gentamicin until the desired time points were reached, when cells were either (i) lysed with 0.5% Triton X-100–PBS and viable counts were performed or (ii) fixed for imaging. XtremeGene 9 (Roche) was used for transfection of HeLa cells following the manufacturer protocols. Lpd RNA interference (RNAi) was performed using Interferin (Polyplus)-mediated delivery of Lpd-specific RAPH1 small interfering RNA (siRNA) (Thermo Scientific). For inhibiting actin polymerization, cytochalasin D (Sigma) was added to culture dishes to reach a 100 ng/ml final concentration at 2 h postinfection as previously described (27), with an equivalent volume of dimethyl sulfoxide (DMSO) without cytochalasin D added to the untreated cells as a control.

### Immunofluorescence imaging.

For immunofluorescence analysis, HeLa cells were grown on glass coverslips and infected at the appropriate multiplicity of infection (MOI), fixed in 3% (wt/vol) paraformaldehyde–PBS for 20 min at room temperature, and permeabilized with 0.1% Triton X-100–PBS. Cells were stained for 20 min with primary and secondary antibodies (Abs) diluted in 0.5 mg/ml bovine serum albumin (BSA)–PBS. L. monocytogenes cells were visualized either through transformation with pJEBAN6 or pNF8 fluorescent vector (28) or by staining with rabbit anti-listeria at a 1:200 dilution and sheep anti-rabbit-Texas Red or goat anti-rabbit-Cy5 (Abcam) at a 1:500 dilution. F-actin was stained with fluorescein isothiocyanate (FITC) or Atto 594-conjugated phalloidin (Sigma) at a 1:400 dilution. DNA was stained with DAPI (4′,6-diamidino-2-phenylindole) (Sigma) at 0.5 μg/ml. Lpd was stained with rabbit anti-LPD serum (from F. B. Gertler, MIT, USA) at a 1:250 dilution with anti-rabbit secondary Ab. VASP was labeled using mouse anti-VASP at 1:100 and donkey anti-mouse-Alexa Fluor 488 (Abcam) at 1:800 dilution. Images were collected on an Olympus BX51 upright microscope using a 60×/0.30 Plan Fln objective and captured using a Coolsnap ES camera (Photometrics) and MetaVue software (Molecular Devices). Specific band pass filter sets for DAPI, FITC, Texas Red, and Cy5 were used to prevent bleed-through from one channel to the next. For live imaging, actin-GFP or actin-red fluorescent protein (RFP) (Invitrogen) was delivered into HeLa cells by the use of a BacMam system to visualize cell actin 24 h before infection. Images were collected on a Swept Field confocal microscope (Nikon) using a 60×/1.40 Plan Apo objective on an TE2000 inverted microscope equipped with the perfect focus system to eliminate focus drift. The settings were as follows: pinholes, 30-μm diameter; scan speed, 1,400 Hz or 100 fps; format, 512 by 512 pixels. GFP and DsRed were subjected to excitation with the 488-nm and 543-nm laser lines, respectively. Images were acquired on a Cascade 512B EM charge-coupled-device (CCD) camera (Photometrics) using Elements software (Nikon) and processed and analyzed using ImageJ (http://rsb.info.nih.gov/ij). The curvature ratio of motile L. monocytogenes is the total distance traveled by each bacterium divided by the start-to-finish distance of its trajectory path. The relative fluorescence intensity (RFI) around each bacterium was retrospectively analyzed by subtracting the integrated intensity of a selected L. monocytogenes-containing area from the area size multiplied by the mean integrated intensity of the background.

### Plaque assay.

Fully confluent monolayers of HeLa cells were infected with the appropriate strain of L. monocytogenes at 2.5 × 10^2^ bacteria per well. Following 2 h of incubation at 37°C, cells were washed and overlaid with 2 ml serum-free DMEM containing 1% Sea-Plaque agarose (Lonza) and 10 μg/ml gentamicin per well and were allowed to set at room temperature. Incubation was continued at 37°C with 5% CO_2_ for 3 to 5 days for plaques to develop.

### Western blot analysis.

Western blot analyses were performed using cytoplasmic extracts subjected to SDS-PAGE. Confluent monolayers of HeLa cells were washed twice with PBS and subsequently scraped in a 250-μl volume of ice-cold NP-40 buffer containing 50 mM Tris-HCl, 200 nM NaCl, 1% Triton X-100, 2 mM MgCl_2_, 10% glycerol, and a 1/20 final volume of protease inhibitor cocktail (Sigma). Following 15 min of incubation on ice, cell lysates were cleared by centrifugation at 16,000 × *g* at 4°C and supernatants were subjected to SDS-PAGE using an 8% polyacrylamide gel and transferred to a nitrocellulose membrane. Lpd was probed with rabbit anti-LPD serum (F. B. Gertler, MIT, USA) at 1:1,000 dilution and IRDye 800CW donkey anti-rabbit at 1:7,500 dilution (Li-COR Biosciences). Monoclonal anti-α-tubulin raised in mouse (Sigma) at 1:1,000 dilution and IRDye 680LT goat anti-mouse secondary antibody (Li-COR Biosciences) at 1:10,000 dilution were used as controls. Images were developed using an Odyssey quantitative fluorescent imaging system (Li-COR Biosciences).

### Statistics.

Statistical analyses were performed using Student's *t* test where *P* < 0.05 indicated a statistically significant difference.

## RESULTS

### Lpd is accumulated with actin-associated L. monocytogenes in an ActA-dependent manner.

**FIG 1:**
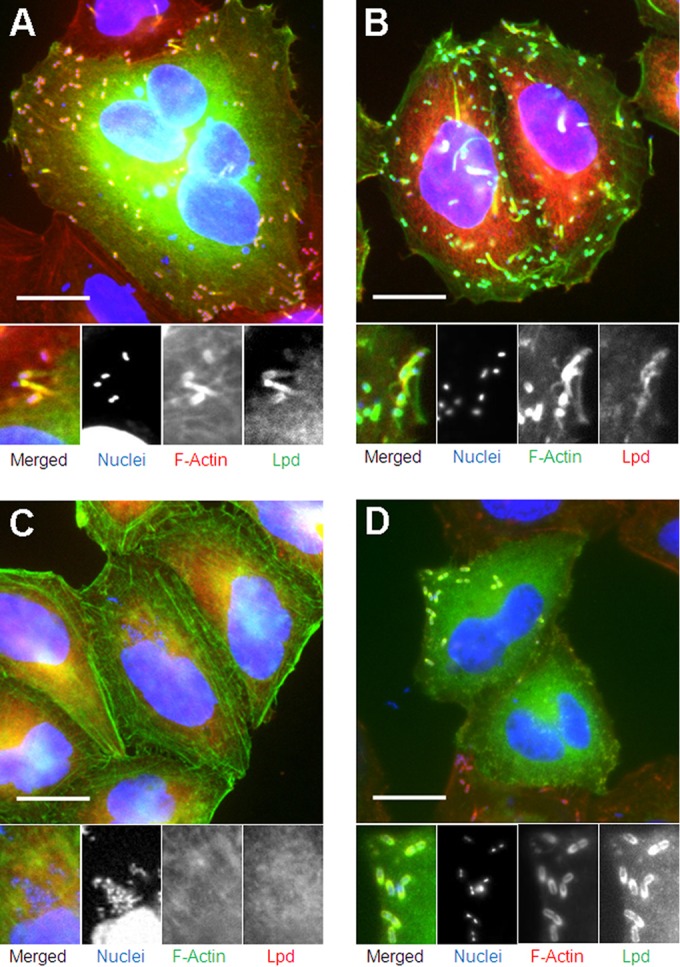
Lpd colocalized with L. monocytogenes in HeLa cells in an ActA-dependent manner. Scale bar = 20 μm. (A) EGFP-tagged Lpd colocalized with some of the actin-associated L. monocytogenes and comet tails 6 h postinfection of HeLa cells. HeLa cells expressing EGFP-tagged Lpd (green) were infected with L. monocytogenes and fixed at 6 h postinfection. Bacteria and HeLa cell nuclei were stained with DAPI (blue). Actin was labeled with phalloidin-Atto 594 (red). (B) Colocalization of Ab-stained Lpd with some of the actin-associated L. monocytogenes and comet tails 6 h postinfection of HeLa cells. HeLa cells were infected with L. monocytogenes and fixed at 6 h postinfection. Bacteria and HeLa cell nuclei were stained with DAPI (blue). Actin and Lpd were labeled with phalloidin-FITC (green) and anti-Lpd (red) Ab, respectively. (C) The L. monocytogenes *ΔactA* strain did not colocalize with F-actin or Lpd in HeLa cells 6 h postinfection. HeLa cells were infected with the L. monocytogenes *ΔactA* strain and fixed at 6 h postinfection. Bacteria and HeLa cell nuclei were stained with DAPI (blue). Actin and Lpd were labeled with phalloidin-FITC (green) and anti-Lpd (red) Ab, respectively. (D) Lpd colocalized with actin-coated L. monocytogenes 6 h postinfection of HeLa cells with inhibited actin polymerization. HeLa cells expressing EGFP-tagged Lpd (green) were infected with L. monocytogenes. Cytochalasin D was added to the cell to reach a concentration of 100 ng/ml at 2 h postinfection, and cells were fixed at 6 h postinfection. Bacteria and HeLa cell nuclei were stained with DAPI (blue). Actin was labeled with phalloidin-Atto 594 (red).

**FIG 2:**
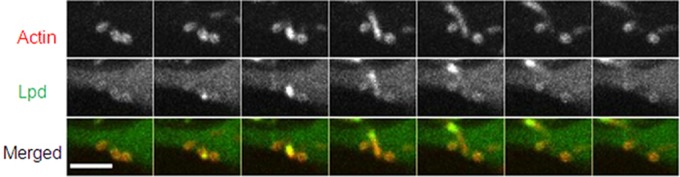
Time lapse live-cell imaging of L. monocytogenes-infected HeLa cells. Live imaging of L. monocytogenes-infected HeLa cells expressing EGFP-tagged Lpd (green) 6 h postinfection. Host actin was labeled with RFP (red). The time lapse between frames was 10 s. Scale bar = 10 μm.

To determine if Lpd was associated with L. monocytogenes at later time points following infection of HeLa cells, HeLa cells were transfected with pEGFP-C1hLPD encoding GFP-tagged Lpd 24 h prior to infection. No Lpd was detectable at 4 h postinfection, in keeping with previous results (23) (data not shown). However, Lpd was observed to accumulate either on the surface of L. monocytogenes or on the actin tails from 6 h postinfection (Fig. 1A). Colocalization was also observed in infected HeLa cells stained with specific anti-Lpd antibody (Fig. 1B), confirming the GFP-Lpd data. The anti-Lpd antibody does not react with L. monocytogenes (data not shown). The association of Lpd with L. monocytogenes was correlated with the recruitment of actin, with 82.23% ± 5.61% of the actin-associated bacteria also having Lpd (calculated from 807 bacteria in 23 cells). In contrast, no Lpd association was observed with intracellular bacteria without actin (11.25% actin-free bacteria among 807 L. monocytogenes bacteria in 23 cells were counted, and 100% were not associated with Lpd) (see Fig. S1 in the supplemental material). Deletion of *actA* led to a complete loss of Lpd colocalization (Fig. 1C), while complementation of *actA* restored the colocalization of Lpd with the bacteria (see Fig. S2). In infected HeLa cells treated with cytochalasin D, which inhibited the formation of F-actin tails, Lpd was still recruited to the surface of L. monocytogenes in 97.02% ± 3.53% of the actin-associated bacteria (calculated from 333 bacteria in 10 cells), predominantly to one pole of the cell (Fig. 1D). To study in more detail the association of Lpd and the initiation of actin-based motility, live imaging of L. monocytogenes-infected HeLa cells expressing GFP-tagged Lpd was conducted 6 h postinfection. We observed the accumulation of Lpd at the interface between the bacteria and the developing actin tail at the initiation of actin-based movement (Fig. 2). This was observed in all bacteria identified to be initiating actin-based movement (26 bacteria in 5 cells).

### Colocalization of Lpd with L. monocytogenes is mediated through PI(3,4)P_2_, VASP, and F-actin.

**FIG 3:**
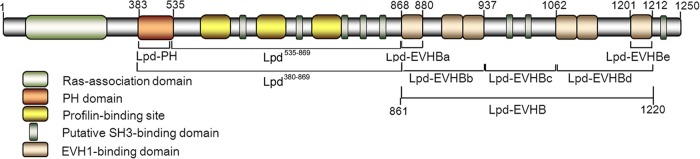
Schematic picture of the domain structure of Lpd. The different functional domains of Lpd are shown. The horizontal lines denote the different constructs fused to the EGFP tag to establish the domains of Lpd important for mediating interactions with L. monocytogenes.

**FIG 4:**
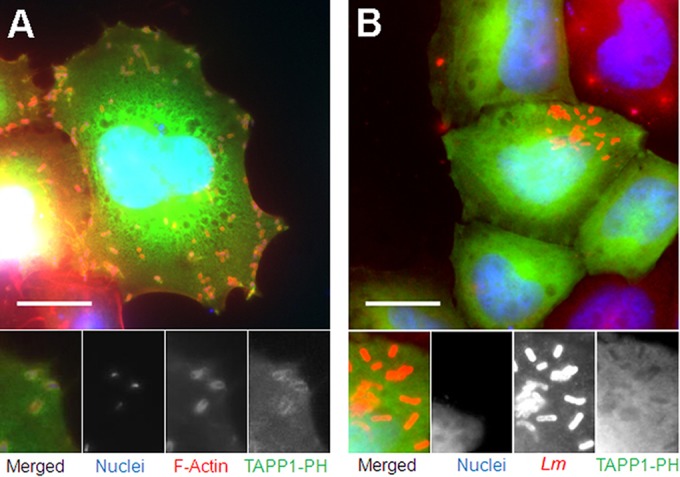
PI(3,4)P_2_ colocalized with L. monocytogenes in HeLa cells in an ActA-dependent manner. Scale bar = 20 μm. (A) The EGFP-tagged TAPP1-PH domain colocalized with actin-associated L. monocytogenes 6 h postinfection of HeLa cells. HeLa cells expressing the EGFP-tagged TAPP1-PH domain (green) were infected with L. monocytogenes and fixed at 6 h postinfection. Bacteria and HeLa cell nuclei were stained with DAPI (blue). Actin was labeled with phalloidin-Atto 594 (red). (B) The EGFP-tagged TAPP1-PH domain did not colocalize with the L. monocytogenes *ΔactA* strain 6 h postinfection of HeLa cells. HeLa cells expressing the EGFP-tagged TAPP1-PH domain (green) were infected with the L. monocytogenes *ΔactA* strain and fixed at 6 h postinfection. Bacteria and HeLa cell nuclei were stained with DAPI (blue). Bacteria were stained with anti-Listeria Ab (red).

Lpd has a multidomain structure (29) (Fig. 3) in keeping with a protein that interacts with a number of effectors (22, 30). Therefore, it is possible that a number of components could mediate the interaction between Lpd and L. monocytogenes. The presence of a PH domain within Lpd (Fig. 3) that recognizes PI(3,4)P_2_ (29) raises the possibility that this lipid could be acting to mediate interactions between Lpd and L. monocytogenes. Although it is known that infection of Vero cells by L. monocytogenes exploiting the InlB-invasion pathway results in increased intracellular levels of PI(3,4)P_2_ (31), no specific association of PI(3,4)P_2_ with intracellular L. monocytogenes has been shown following InlB-Met receptor-mediated invasion of HeLa cells (reviewed in reference 32). Therefore, to establish if PI(3,4)P_2_ plays any role in mediating interactions, we first needed to ascertain if there was a specific interaction between PI(3,4)P_2_ and intracellular L. monocytogenes. To detect such an interaction, 24 h prior to L. monocytogenes infection, HeLa cells were transfected with a plasmid expressing the GFP-TAPP1-PH domain fusion which specifically binds to PI(3,4)P_2_—and not to other phosphoinositides (33). Subsequently, we observed that PI(3,4)P_2_ accumulated on the surface of 68.15% ± 9.35% (*P* = 0.0172 compared to Lpd) of the actin-associated L. monocytogenes bacteria as well as on 82.26% ± 10.48% of the actin tails from 6 h postinfection (calculated from 492 bacteria in 11 cells) (Fig. 4A). This is the first demonstration of a specific association of the lipid with intracellular L. monocytogenes. The colocalization of PI(3,4)P_2_ with L. monocytogenes was dependent on the *Listeria* ActA protein, since colocalization of GFP-TAPP1-PH with L. monocytogenes was no longer observed in an Δ*actA* mutant (Fig. 4B).

**FIG 5:**
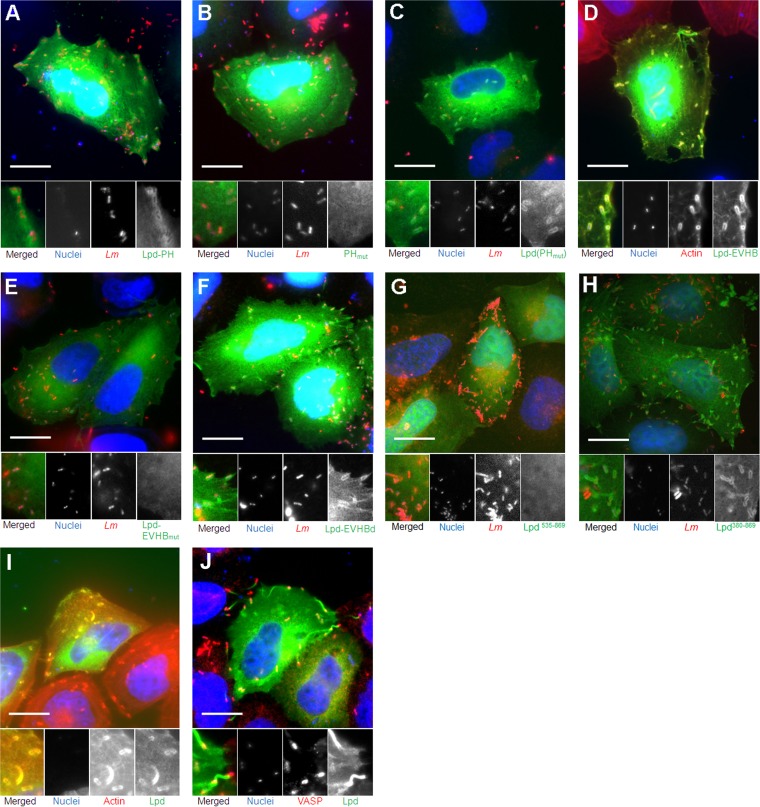
Colocalization of Lpd and Lpd derivatives with L. monocytogenes 6 h postinfection of HeLa cells. (A) The EGFP-tagged Lpd-PH domain colocalized with L. monocytogenes 6 h postinfection of HeLa cells. HeLa cells expressing the EGFP-tagged Lpd-PH domain (green) were infected with L. monocytogenes (pJEBAN6) (red) and fixed at 6 h postinfection. Bacteria and HeLa cell nuclei were stained with DAPI (blue). Scale bar = 20 μm. (B) The EGFP-tagged mutated Lpd-PH domain, which abolished PI(3,4)P_2_ binding, did not colocalize with L. monocytogenes 6 h postinfection of HeLa cells. HeLa cells were transfected with pEGFP-Lpd-PH_mut_ and infected with L. monocytogenes (pJEBAN6). Bacteria and HeLa cell nuclei were stained with DAPI. Scale bar = 20 μm. (C) EGFP-tagged Lpd mutated in the PH domain, which abolished PI(3,4)P_2_ binding, still colocalized with L. monocytogenes 6 h postinfection of HeLa cells. HeLa cells expressing EGFP-tagged Lpd(PH_mut_) (green) were infected with L. monocytogenes (pJEBAN6) (red) and fixed at 6 h postinfection. Bacteria and HeLa cell nuclei were stained with DAPI (blue). Scale bar = 20 μm. (D) The EGFP-tagged Lpd-EVH1 binding domain colocalized with actin-associated L. monocytogenes 6 h postinfection of HeLa cells. HeLa cells expressing the EGFP-tagged Lpd-EVHB domain (green) were infected with L. monocytogenes and fixed at 6 h postinfection. Bacteria and HeLa cell nuclei were stained with DAPI (blue). Actin was labeled with phalloidin-Atto 594 (red). Scale bar = 20 μm. (E) Disruption of all five EVH1 binding domains in the EVH1cy region of Lpd abolished colocalization of Lpd with L. monocytogenes 6 h postinfection of HeLa cells. HeLa cells expressing the EGFP-tagged Lpd-EVHB_mut_ domain (green) were infected with L. monocytogenes (pJEBAN6) (red) and fixed at 6 h postinfection. Bacteria and HeLa cell nuclei were stained with DAPI (blue). Scale bar = 20 μm. (F) Lpd-EVHBd-EGFP construct that contains the three C-terminal EVH1 binding domains colocalized with L. monocytogenes 6 h postinfection of HeLa cells. HeLa cells expressing the EGFP-tagged Lpd-EVHBd domain (green) were infected with L. monocytogenes (pJEBAN6) (red) and fixed at 6 h postinfection. Bacteria and HeLa cell nuclei were stained with DAPI (blue). Scale bar = 20 μm. (G) EGFP-tagged Lpd without the PH domain or EVHB region did not colocalize with L. monocytogenes 6 h postinfection of HeLa cells. HeLa cells expressing EGFP-tagged Lpd^535–869^ (green) were infected with L. monocytogenes (pJEBAN6) (red). Bacteria and HeLa cell nuclei were stained with DAPI (blue). Scale bar = 20 μm. (H) EGFP-tagged Lpd without the EVHB region colocalized with L. monocytogenes 6 h postinfection of HeLa cells. HeLa cells expressing EGFP-tagged Lpd^380–869^ (green) were infected with L. monocytogenes (pJEBAN6) (red). Bacteria and HeLa cell nuclei were stained with DAPI (blue). Scale bar = 20 μm. (I) Mutating the EVH1 binding region in L. monocytogenes ActA resulted in significantly less Lpd recruitment to the surface of the bacteria. HeLa cells expressing EGFP-tagged Lpd (green) were infected with the *L. monocytogenes ΔactA*^*EVHB*^ strain and fixed at 6 h postinfection. Bacteria and HeLa cell nuclei were stained with DAPI (blue). Actin was labeled with phalloidin-Atto 594 (red). Scale bar = 20 μm. (J) VASP did not locate on the actin tails of L. monocytogenes. HeLa cells expressing EGFP-tagged Lpd were infected with wild-type (WT) L. monocytogenes and fixed at 6 h postinfection. Bacteria and HeLa cell nuclei were stained with DAPI (blue). VASP was labeled with anti-VASP antibody. Scale bar = 20 μm.

To elucidate in more detail the mechanism of Lpd colocalization with intracellular L. monocytogenes, truncated versions of EGFP-tagged Lpd were expressed in HeLa cells prior to infection with the bacterium (Fig. 3). The results showed that the PH domain of Lpd alone, which specifically recognizes PI(3,4)P_2_, was colocalized with 66.50% ± 12.46% of actin-associated L. monocytogenes bacteria [calculated from 205 bacteria in 8 cells; *P* > 0.05 compared to PI(3,4)P_2_] (Fig. 5A). When the PH domain was disrupted by site-directed mutagenesis [changes K414A and R416A, which are basic amino acids known to be essential for PI(3,4)P_2_ binding], the resultant domain, PH_mut_, lost its ability to interact with L. monocytogenes (Fig. 5B), confirming that this interaction was mediated via PI(3,4)P_2_. However, the same mutation in the full-length Lpd protein, Lpd(PH_mut_)-EGFP, did not show any significant difference in colocalization with L. monocytogenes (Fig. 5C). These data demonstrate that association of Lpd with L. monocytogenes may involve additional interactions beside those mediated by the PH domain.

One possibility is that Lpd could be interacting via its EVH1 binding domains (Fig. 3) with VASP bound to the L. monocytogenes cell surface by ActA. To establish whether interactions between VASP and Lpd are important, the C-terminal half of Lpd, in which the six EVH1 binding domains are located, was fused to EGFP to generate Lpd-EHVB-EGFP (Fig. 3). This construct still recruited VASP (data not shown) and mediated levels of colocalization with L. monocytogenes comparable to those seen with the full-length Lpd (colocalization with 77.80% ± 8.33% of actin-associated L. monocytogenes 6 h postinfection, calculated from 387 bacteria in 10 cells; *P* > 0.05 compared to Lpd) (Fig. 5D). Disruption of all five EVH1 binding domains and hence of EVH1 binding ability by substituting the Phe or Leu in all of the six FPPPP/LPPPP domains (34) in Lpd-EVHB-EGFP to generate Lpd-EVHB_mut_-EGFP abolished binding to VASP (data not shown) and colocalization of the EVHB region with L. monocytogenes (Fig. 5E). This interaction was further refined by making truncated fusions of the EVHB region to EGFP (Fig. 3). In essence, the only truncated construct capable of mediating interactions with L. monocytogenes comparable to that seen with full-length Lpd was EVHBd-EGFP, which contains the three C-terminal EVH1 binding domains (colocalized with 78.73% ± 5.25% of actin-associated L. monocytogenes bacteria, calculated from 413 bacteria in 10 cells; *P* > 0.05 compared to Lpd and Lpd-EVHB) (Fig. 5F). To confirm that colocalization of Lpd with L. monocytogenes was mediated by both the PH domain and the EVH-1 domain, two further constructs were made. First, the central portion of Lpd from position 535 to position 869, which lacks both the PH and EVH1 binding domains (Fig. 3), was fused to EGFP. Second, a fusion of Lpd from position 380 to position 869, which includes the PH domain (Fig. 3), to EGFP was made. The absence of both the PH and EVH1 binding domains abolished colocalization with L. monocytogenes (Fig. 5G), whereas, in contrast, the presence of the PH domain resulted in colocalization (Fig. 5H) (86.25% ± 3.86%, calculated from 302 bacteria in 10 cells). Based on these observations, we proposed that the colocalization of Lpd with L. monocytogenes was mediated via binding to both PI(3,4)P_2_ and the VASP protein, which is recruited to the surface of L. monocytogenes by ActA (34).

**FIG 6:**
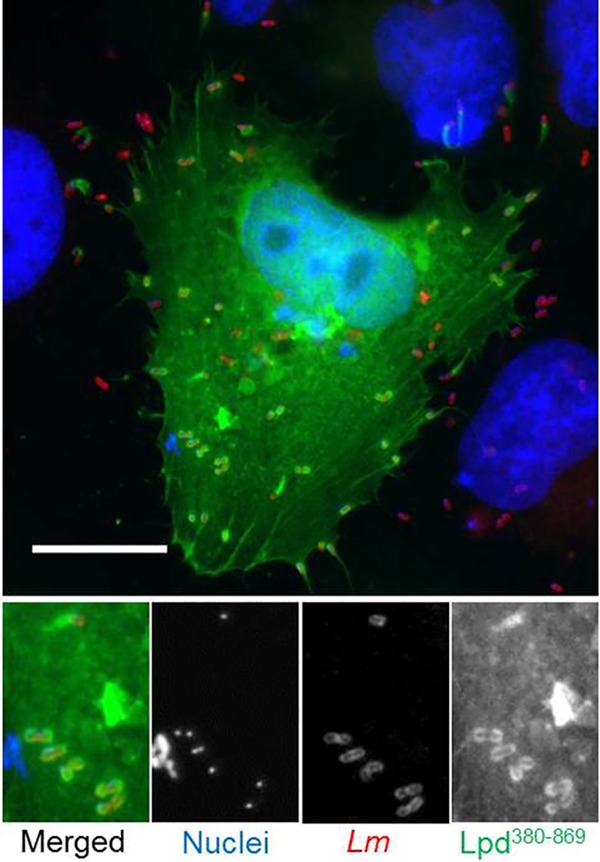
Colocalization of Lpd^380–869^ with L. monocytogenes *ΔactA*^*EVHB*^ 6 h postinfection of HeLa cells. HeLa cells expressing EGFP-tagged Lpd^380–869^ (green) were infected with L. monocytogenes *ΔactA*^*EVHB*^ and fixed at 6 h postinfection. Nuclei were stained with DAPI (blue). Bacteria were stained with anti-Listeria Ab (red). Scale bar = 20 μm.

In order to test whether VASP was absolutely required for Lpd colocalization, an L. monocytogenes *actA* mutant was generated. This strain was deleted for amino acids 264 to 390 (Δ*actA*^*EVHB*^), which is the EVH1 binding region (34). This strain (the Δ*actA*^*EVHB*^ strain) could no longer bind VASP and hence formed thinner and shorter tails (see Fig. S3 in the supplemental material) and was also significantly attenuated in actin-based movement, with less and smaller plaque formation (*P* = 0.0135, *n* = 3). The strain recruited less Lpd to its surface, but some Lpd was still recruited to the actin tails (compare Fig. 1A and B and 5I). Since there was no VASP recruitment to the Δ*actA*^*EVHB*^ strain (see Fig. S3), the association of Lpd with the bacteria could possibly have been mediated through PI(3,4)P_2_. To confirm this, the localization of the aa-380-to-869 construct of Lpd that retains the PH domain but lacks the EVH1 domain (Fig. 3) was studied in cells infected with the Δ*actA*^*EVHB*^ strain. This truncated construct of Lpd still colocalized with the Δ*actA*^*EVHB*^ strain (Fig. 6), supporting the hypothesis that colocalization is mediated through PI(3,4)P_2_ in the absence of VASP. Although VASP has been reported to bind directly to the barbed end of actin filaments and compete with capping protein for the continued growth of actin filaments (35), anti-VASP staining failed to locate VASP on the actin tails of L. monocytogenes (Fig. 5J). Therefore, one possibility is that Lpd can bind directly to F-actin through another as-yet-unknown mechanism.

### Lpd is involved in the cell-to-cell spread and the initiation of actin-based movement of L. monocytogenes.

**FIG 7:**
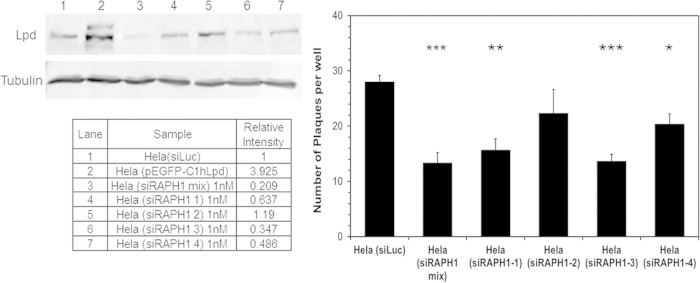
Depletion of Lpd in HeLa cells using RAPH1 siRNA interference significantly reduced the number of plaques formed by L. monocytogenes. The results of Western blot analysis using anti-Lpd antibody against the cell lysate of HeLa cells either transformed with Lpd-expressing plasmid or transfected with 1 nM nonspecific Luc siRNA as a control with RAPH1 individual or mixed siRNAs for 3 days are shown. Tubulin was used as the control. The relative intensity of the band of each sample against HeLa cell lysate was measured using ImageJ. The number of plaques was significantly lower in HeLa cell knockdown of Lpd expression using a RAPH1 siRNA mixture and three of the individual siRNAs. Error bars indicate standard errors. Stars indicate statistically significant differences. *, *P* < 0.05; **, *P* < 0.005; ***, *P* < 0.0005; *n* = 3.

Based on the role of protrusions in the cell-to-cell spread of L. monocytogenes and the function of Lpd in lamellipodin formation, we decided to investigate the role of Lpd in the dissemination of L. monocytogenes between cells using a plaque assay. In this assay, the cell-to-cell spread is visualized by the presence and size of plaques in a lawn of cells (27). Lpd expression was depleted in HeLa cells by RNAi, using four independent Lpd-specific siRNAs. Western blot analysis demonstrated that a mixture of siRNAs significantly depleted the levels of Lpd, as did three individual siRNAs (Fig. 7). Cells infected with L. monocytogenes with decreased Lpd expression showed significantly fewer and smaller plaques, with a correlation between the level of Lpd depletion and the numbers of plaques formed (Fig. 7). This is the first evidence of a role for Lpd in the cell-to-cell spread of L. monocytogenes.

The role that Lpd played in bacterium protrusion was tested by calculating the percentages of L. monocytogenes-containing protrusions (listeriopods) observed among all actin-associated bacteria in HeLa cells, HeLa cells knocked down for expression of Lpd, and HeLa cells overexpressing Lpd on the basis of the results of live-cell imaging in which 4 to 8 films were analyzed. In HeLa cells, the protrusion ratio was 29.48% among all actin-associated bacteria. In HeLa cells knocked down for expression of Lpd by siRNA, the number was 20.58%, which was not significantly different from that in the other HeLa cells. However, in cells overexpressing Lpd, the protrusion ratio was 47.67%, which was significantly higher than that in HeLa cells (*P* = 0.0278) and in HeLa cells knocked down for Lpd (*P* = 0.00328).

### Lpd concentration affects the speed and direction of L. monocytogenes motility.

To establish that the decrease in cell-to-cell spread detected in the plaque assay in cells with reduced Lpd expression was not a consequence of the reduction in Lpd affecting the intracellular velocity of L. monocytogenes, live-cell imaging was performed to measure directly the actin-based motility in HeLa cells either overexpressing or depleted of Lpd (see Videos S1 to S3 in the supplemental material). The result showed that there was a significantly higher percentage of motile bacteria among all actin-associated L. monocytogenes bacteria in HeLa cells overexpressing EGFP-Lpd (68%) than in wild-type HeLa cells (only 22%) (*P* = 2.45 × 10^−5^, *n* = 6). In contrast, the depletion of Lpd via siRNA did not significantly affect this ratio, with 28% of the actin-coated bacteria being motile compared to 22% in wild-type HeLa cells (*P* = 0.086, *n* = 6). However, while increased expression of EGFP-Lpd in HeLa cells did lead to a reduction in tail length (*P* = 4.75 × 10^−10^, *n* = 10) and increased directional alteration (*P* = 0.00357, *n* = 30), there was no significant difference in the intracellular velocity of L. monocytogenes, which was 20.1 ± 2.6 μm/min in HeLa cells compared to 17.5 ± 2.2 μm/min in HeLa cells expressing EGFP-Lpd (see Videos S1 and S2). In contrast, depleting Lpd resulted in an increased velocity of 36.4 μm/min, which was significantly different from that seen in wild-type HeLa cells (*P* = 4.91 × 10^−11^, *n* = 30). However, tail length or directional persistence did not significantly change (see Videos S1 and S3). As such, the reduction in cell-to-cell spread observed when Lpd was depleted cannot be explained by a reduction in the intracellular velocity of the L. monocytogenes bacteria (see Videos S1 to S3). The effects on motility and intracellular velocity of L. monocytogenes as a result of changes in the levels of Lpd therefore suggest that Lpd has other important roles during the infection cycle.

## DISCUSSION

In this study, we made a number of critical discoveries that provide new insights into the cytosolic life cycle of L. monocytogenes and its transmission from cell to cell. We discovered that Lpd could be detected on the surface of L. monocytogenes 6 h postinfection and that Lpd is involved in cell-to-cell spread and actin-based motility. Analysis of the interaction between Lpd and L. monocytogenes revealed that a number of factors are involved. The discovery that PI(3,4)P_2_ was detectable on the surface of intracellular L. monocytogenes 6 h postinfection suggested that PI(3,4)P_2_ might play a role in mediating the interaction between L. monocytogenes and Lpd. The observation that site-directed mutagenesis of the PH domain in Lpd, which recognizes PI(3,4)P_2_, failed to abolish or diminish the interaction between Lpd and L. monocytogenes indicated that interaction via the lipid PI(3,4)P_2_ route on the bacterial cell surface cannot be the only route by which this interaction is mediated. Indeed, it has recently been shown that Lpd binds PI(3,4)P_2_ with low affinity (29). Structure function analysis of Lpd indicated that the EVH1 binding domains of Lpd were critical in mediating interactions between Lpd and L. monocytogenes (Fig. 5E). While one must be cautious to avoid overinterpreting experiments in which truncated fragments of proteins are expressed, the data clearly show that this interaction is mediated predominantly by the three EVH1 domains in the C terminus of Lpd. The observation that disruption of VASP binding to L. monocytogenes by deletion of the EVH1 binding domain in ActA (Fig. 5I) also reduced Lpd binding to the bacterium is in keeping with a hypothesis that Lpd interacts with L. monocytogenes by interaction with VASP via EVH1 binding domains in Lpd. However, Lpd could also be detected in the actin tails, where VASP could not be detected (Fig. 5J), suggesting that Lpd can also interact directly with polymerized actin through an as-yet-unidentified mechanism.

Live-cell imaging clearly showed that strong accumulation of Lpd at the initiation of the movement of bacteria and overexpression of Lpd increased the percentage of motile bacteria. Since high-affinity Ena/VASP-ActA binding is important for early initiation of actin-based motility (17), Lpd may function in either increasing the VASP concentration at the bacterial surface or increasing the binding affinity of VASP with ActA. This model could also explain the changes in bacterial mobility we observed in HeLa cells overexpressing or depleted for Lpd. Lpd was reported to facilitate locating VASP protein to the lamellipodial tip of the plasma membrane (23, 36). As such, in Lpd knockdown cells, VASP protein would no longer be concentrated on the plasma membrane through association with Lpd, thereby raising the levels of cytosolic VASP. As the VASP concentration greatly affects the speed of L. monocytogenes movement (17), this could explain the increased moving speed of L. monocytogenes in Lpd knockdown cells. When Lpd was overexpressed, excess Lpd competed with L. monocytogenes ActA for the available VASP binding, leading to decreased directional persistence and a shorter tail length, a phenotype similar to that seen with reduced levels of VASP expression in the cell.

The Lpd knockdown experiment showed that the level of intracellular Lpd was in direct proportion to the efficiency of L. monocytogenes cell-to-cell spread, suggesting that Lpd was required for the spreading of L. monocytogenes. In Lpd knockdown HeLa cells, the velocity of L. monocytogenes movement increased; therefore, the attenuation in cell-to-cell spread was probably due not to impaired L. monocytogenes movement but to attenuation in filopod formation and L. monocytogenes protrusion. As Lpd was required for formation of lamellipodia and filapodia, when L. monocytogenes reaches the periphery of the host cell, Lpd attracted to L. monocytogenes may help the formation of protruding L. monocytogenes-containing filopods.

It has been reported that several mechanisms contribute to the protrusion of motile L. monocytogenes. One is the secretion of L. monocytogenes InlC protein, which physically interacts with and antagonizes the function of a human cytoplasmic protein, Tuba. InlC binds to the SH3 domain of Tuba, blocking its interaction with N-WASP, and releases the cortical tension in cell junctions (19, 37, 38). It has recently been shown that spreading of L. monocytogenes involves antagonism of the GTPase Cdc42 and that this may involve impairment of Tuba (20). The other factor is the host cytoskeletal regulatory protein ezrin, a member of the ERM family (18). ERM proteins were previously suggested to confer rigidity to L. monocytogenes protrusions by cross-linking F-actin tails to the host plasma membrane (37). Both factors contribute to L. monocytogenes cell-to-cell spread by releasing the inward tensions at cell junctions. Is it very likely that Lpd plays a similar role in remodelling actin dynamics at the cell surface to promote protrusions. It has not been investigated whether Lpd interacts with Tuba or Cdc42; however, as Lpd also contains 8 SH3 binding domains, it is possible that Lpd also binds Tuba or other SH3-containing proteins.

In conclusion, this report identifies Lpd as a new determinant factor in the actin-based mobility and cell-to-cell spread of L. monocytogenes. This report adds to our current understanding of the well-established model of actin-based movement and spreading of L. monocytogenes and sheds new light on the interaction between pathogen and host.

## Supplementary Material

Supplemental material
