# Supplementary material for: Lamellipodin Is Important for Cell-to-Cell Spread and Actin-Based Motility in Listeria monocytogenes
Source: Infect Immun. 2015 Aug 12;83(9):3740–8. doi: 10.1128/IAI.00193-15 (PMC4534642; doi:10.1128/IAI.00193-15)
Supplement: Supplemental material [file supp_83_9_3740__index.html]

Lamellipodin Is Important for Cell-to-Cell Spread and Actin-Based Motility in Listeria monocytogenes — Supplemental material 

# Lamellipodin Is Important for Cell-to-Cell Spread and Actin-Based Motility in Listeria monocytogenes

## Supplemental material

- Supplemental file 1 -

  Table S1. Primer list. Fig. S1. Ab-stained Lpd did not colocalise with *L. monocytogenes* not recruiting actin 6 h postinfection of Hela cells. Fig. S2. Lpd colocalised with the complemented *L. monocytogenes* Δ*actA* strain in Hela cells 6 h postinfection. Fig. S3. The *L. monocytogenes* strain Δ*actA*EVHB could no longer bind VASP, hence forming thinner and shorter tails in Hela cells 6 h postinfection. Legends to Videos S1 to S3.

  PDF, 1.1M
- Supplemental file 2 -

  Video S1. Actin-based intracellular movement of *L. monocytogenes* in Hela cells 6 h postinfection at 50x speed.

  AVI, 2.1M
- Supplemental file 3 -

  Video S2. Actin-based intracellular movement of *L. monocytogenes* in Hela cells overexpressing Lpd 6 h postinfection at 50x speed.

  AVI, 2.4M
- Supplemental file 4 -

  Video S3. Actin-based intracellular movement of *L. monocytogenes* in Hela cells depleted of Lpd expression 6 h postinfection at 50x speed.

  AVI, 1.9M
