# Supplementary material for: Lamellipodin Is Important for Cell-to-Cell Spread and Actin-Based Motility in Listeria monocytogenes
Source: Infect Immun. 2015 Aug 12;83(9):3740–8. doi: 10.1128/IAI.00193-15 (PMC4534642; doi:10.1128/IAI.00193-15)
Supplement: Supplemental material [file IAI.00193-15_zii999091392so1.pdf]

Table S1

Name	Sequence (5'->3')
actAKO1-F	AGATAAGAATTCGGTTATTTTATTGCTCC
actAKO1-R	CGATAAGGATCCGTTTAATCCCACTTATAC
actAKO2-F	CGTGTAGGATCCAATAATTAAAAACACAG
actAKO2-R	ATATCTGGATCCTGCGGTAAAATTAT
actAEVHBKO1-F	CTGAGAATTCACCAAGATACGAAACTGCAC
actAEVHBKO1-R	ATCAGGATCCCGAAGCATTTACCTCTTCAC
actAEVHBKO2-F	ATAAGGATCCAGAGGCGGTAGACCAACATC
actAEVHBKO2-R	ATTGAAGCTTCCAGCAGAACGATTTTTTCC
LPD380-F	TTACTCCTCGAGATGAACAAAGAAGTCCTCTTG
LPD535-F	TTACTCCTCGAGATGAAATCGGGATCCAGTTCT
LPD869-R	TTACTCGAATTCAAACTGGCTGGCTATCTGCTT
PH-SDM-K-F	GATGGCAAGAAGTCCTGGGCCAAGCGTTATTTTCTC
PH-SDM-K-R	GAGAAAATAACGCTTGGCCCAGGACTTCTTGCCATC
PH-SDM-R-F	GAAGTCCTGGGCCAAGGCCTATTTTCTCTTGCGAGC
PH-SDM-R-R	GCTCGCAAGAGAAAATAGGCCTTGGCCCAGGACTT

Primer list.

Fig. S1

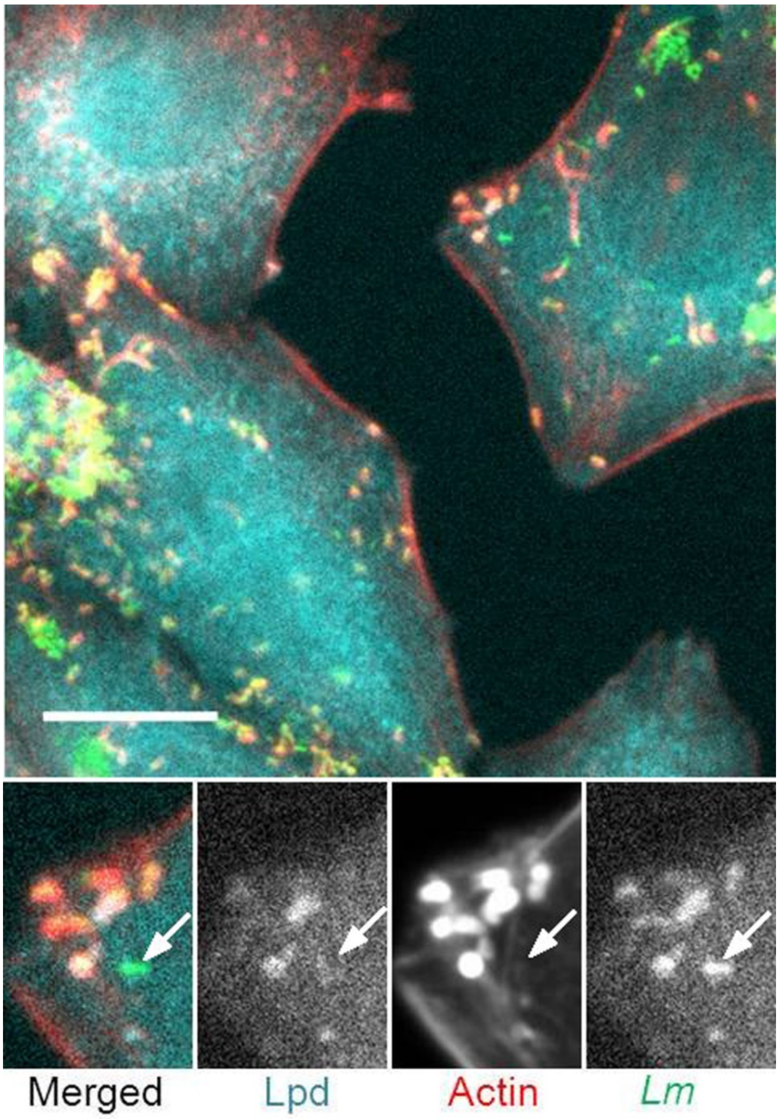

Ab-stained Lpd did not co-localise with *L. monocytogenes* not recruiting actin 6 hr post-infection of Hela cells. Hela cells were infected with *L. monocytogenes* InlAm(pNF8) (green). Actin and Lpd were labelled with Phalloidin-Atto594 (red) and anti-Lpd-Cy5 (Cyan) Ab respectively. Arrows pointed out a bacterium without actin or Lpd association. Scale bar=20μm.

Fig. S2

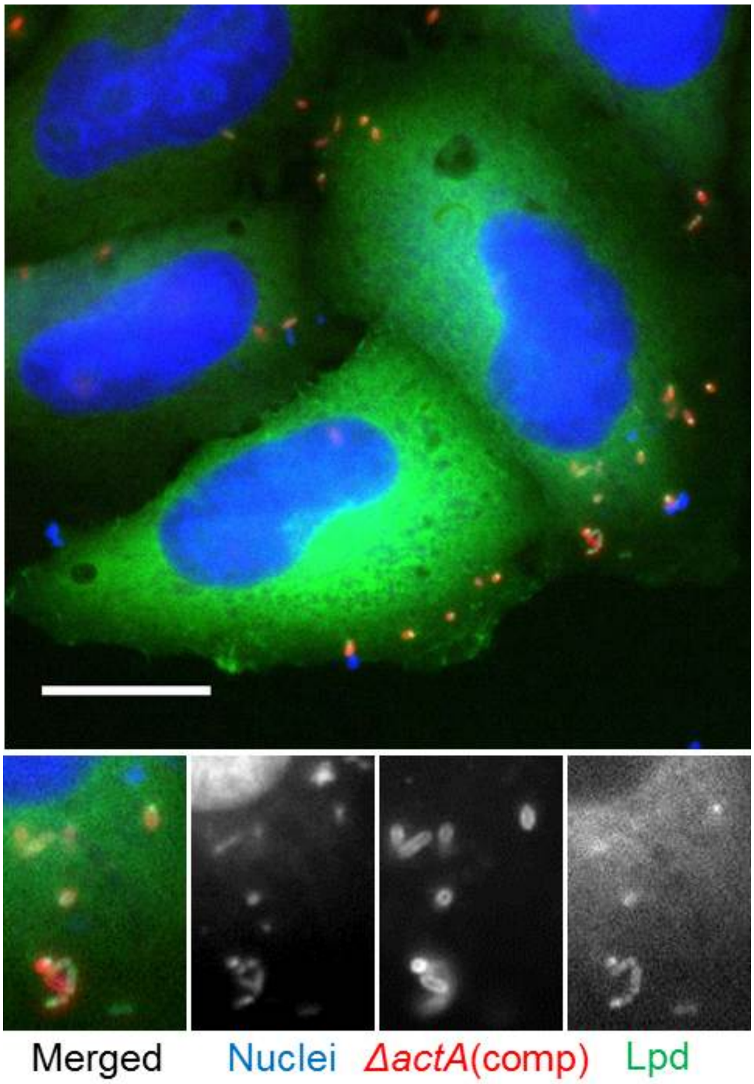

Lpd co-localised with the complemented *L. monocytogenes ΔactA* strain in Hela cells 6 hr post infection. Hela cells were transfected with pEGFP-C1hLpd (green) and infected with *L. monocytogenes ΔactA* strain harbouring the pUNK1-actA plasmid (red) and fixed at 6 hr post infection. Bacteria and Hela cell nuclei were stained with DAPI. Scale bar=20 μm.

Fig. S3

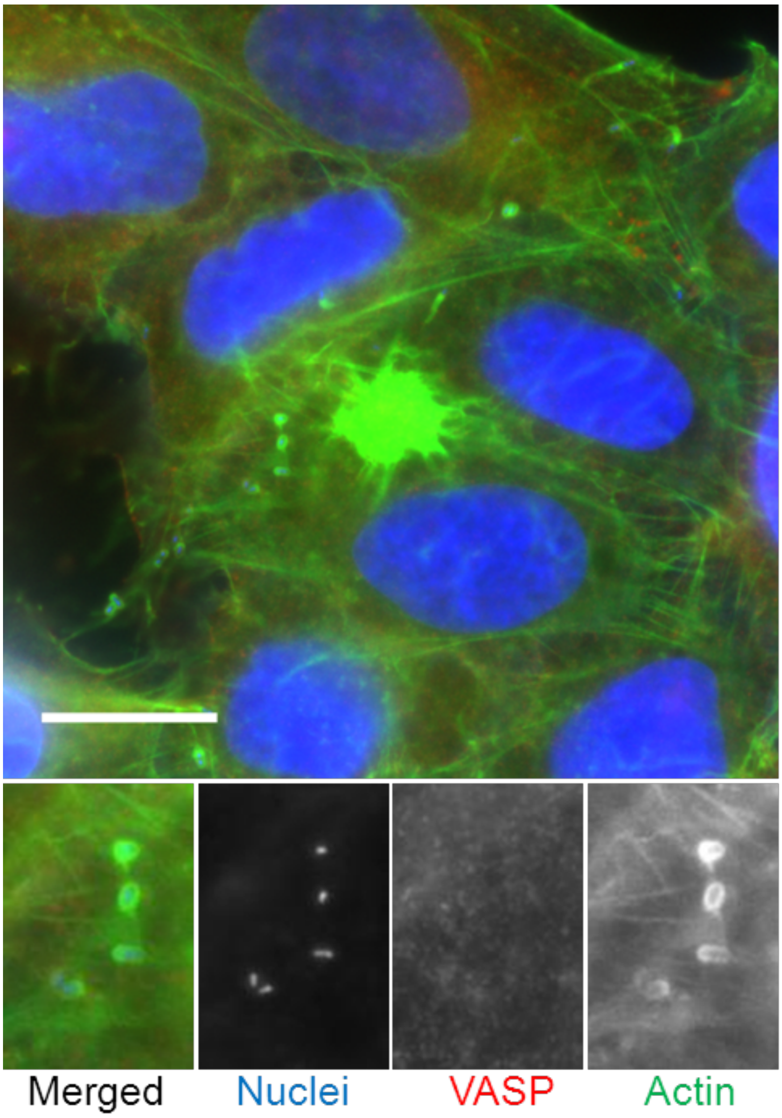

The *L. monocytogenes* strain *ΔactA*^{EVHB} could no longer bind VASP hence forming thinner and shorter tails in Hela cells 6 hr post infection. Actin and VASP were labelled with phalloidin-GFP and anti-VASP Ab. Bacteria and Hela cell nuclei were stained with DAPI. Scale bar=20 μm.

Video S1

The actin-based intracellular movement of *L. monocytogenes* in Hela cells 6 hr post infection 50X speed. Camera captured the signal from labelled F-actin.

Video S2

The actin-based intracellular movement of *L. monocytogenes* in Hela cells over-expressing Lpd 6 hr post infection 50X speed. Hela cells were transfected with pEGFP-C1hLPD 24 hr before infection. Camera captured the signal from labelled F-actin.

Video S3

The actin-based intracellular movement of *L. monocytogenes* in Hela cells depleted of Lpd expression 6 hr post infection 50X speed. Hela cells were transfected with Lpd-specific RAPH1 siRNAs 48 hr before infection. Camera captured the signal from labelled F-actin.
